# ST segment change and T wave amplitude ratio in lead aVR associated with coronary artery disease severity in patients with non-ST elevation myocardial infarction: A retrospective study

**DOI:** 10.1097/MD.0000000000009062

**Published:** 2017-12-08

**Authors:** Yahya Kemal İçen, Mevlüt Koç

**Affiliations:** Department of Cardiology, Adana Health Practices and Research Center, Health Sciences University, Adana, Turkey.

**Keywords:** coronary, lead aVR, NSTEMI, ratio, syntax

## Abstract

Non-ST elevation myocardial infarction (NSTEMI) severity is difficult only with electrocardiogram (ECG). In most cases, NSTEMI patients are followed with cardiac enzymes without early invasive intervention if no severe chest pain exists in the emergency department (ED) or coronary intensive care unit (CICU).

Our aim was to evaluate association between CAD severity and ST segment change in lead aVR (STaVR)/T wave amplitude in lead aVR (TAaVR) ratio in patients with NSTEMI.

We included 306 patients with NSTEMI in the ED between 2015 and 2016. STaVR and TAaVR values were measured from 12-derivation ECG underwent on admission to the ED. The absolute values of STaVR and TAaVR were calculated and the following were obtained; ratio 1:|STaVR|/|TAaVR|, ratio 2:|TAaVR|/|STaVR|, and ratio 3: by dividing the variable with larger absolute value to other variable with smaller absolute value (|larger value|/|smaller value|). The SYNTAX score (SS) was calculated from coronary angiography in all patients.

In analysis of the bivariate correlation between SS and ratios; significantly positive, strongly with ratio 3 (*r* = 0.692, *P* < .001), and only ratio 3 was determined to be an independent predictor for SS in linear regression analysis (OR: 0.642, 95% CI: 0.432–0.853, *P* = .001).

Severity of CAD may be estimated by evaluating STaVR and TAaVR ratio in patients with NSTEMI.

## 1 Introduction

Acute myocardial infarction is divided into 3 groups depending on electrocardiography (ECG) findings including ST elevation myocardial infarction (STEMI), non-ST elevation myocardial infarction (NSTEMI), and bundle branch block myocardial infarction (BBBMI).^[1]^ Diagnosis of STEMI is easy and inexpensive via a 12-derivation ECG. Since nonspecific ECG findings such as ST depression and negative T wave or no ECG finding is detected in approximately 60% of the patients, NSTEMI diagnosis is difficult only with ECG.^[2]^ In most cases, NSTEMI patients are followed with cardiac enzymes without early invasive intervention if no severe chest pain exists in the emergency department (ED) or coronary intensive care unit (CICU).

The number of the patients with NSTEMI is more than the patients with STEMI, and the prognosis tend to progress badly since coronary artery disease (CAD) is more common and severe.^[3]^ The Synergy Between PCI With Taxus and CABG (SYNTAX) score (SS) is a comprehensive scoring method used to show the incidence of the CAD which is obtained from coronary angiography (CAG) images and depends on lesion location, complexity, and calcification.^[4]^ SS is a predictor for cardiac mortality in the patients with NSTEMI.^[5]^

Recent articles reported that lead aVR would also be useful as precordial and extremity leads.^[6]^ The previous studies showed an association between mortality and T wave amplitude in lead aVR (TAaVR) in the patients with STEMI and NSTEMI.^[7,8]^ In NSTEMI patients, ST depression, negative T wave in derivations indicating anterior and inferior wall, as well as ST segment changes in lead aVR (STaVR) and TAaVR are observed by increase of CAD severity. Although there is a study indicating that positive TAaVR and elevated STaVR affect the prognosis adversely in the patient with NSTEMI,^[8]^ no study about CAD severity with evaluation of both exists. The aim of the present study is to evaluate associations between CAD severity and STaVR/TAaVR ratio.

## 2 Methods

Patient population: Patients who were admitted to CICU due to the diagnosis of NSTEMI in the ED between 2015 and 2016 were included in the present retrospective study. The study was approved by the ethics committee of Çukurova University. Informed consent form was signed by all patients. Such patients were diagnosed with ischemic ST changes, T wave inversion in ECG, and positive cardiac enzymes within 24 hours. The patients with ST elevation, renal function disorders, hepatic function disorder, and previous percutaneous coronary or coronary artery by-pass procedure were excluded. The demographic data of the patients were recorded.

Evaluation of laboratory findings: High sensitivity troponin T (hs-TnT), creatinine kinase myocardial band (CK-MB), renal function tests, lipid parameters, high sensitivity C reactive protein (hs-CRP), uric acid, and whole blood count analyses were recorded from routine blood test analyses.

Electrocardiographic and echocardiographic assessment: The 12-lead surface ECG (Nihon Kohden, Cardiofax V; model ECG-1550K, rate, 25 mm/s and standard 1 mV/10 mm) findings which were obtained after admission to the ED were evaluated by 2 cardiologists individually. QRS duration, and negative and positive numeric data according to the below and above location of ST segment in lead aVR, respectively, were recorded. TAaVR was measured depending on the PR segment in lead aVR so that negative values below segment (<0) and positive numeric values above segment (0≥) and through total vector magnitude for biphasic values. The absolute values of STaVR and TAaVR were subsequently calculated and the following were obtained; ratio 1: |STaVR|/|TAaVR|, ratio 2: |TAaVR|/|STaVR|, and ratio 3: by dividing the variable with larger absolute value by other variables with a smaller absolute value in lead aVR (ratio 3: |larger value|/|smaller value|). Ejection fractions (EF) were also recorded echocardiographically (Phillips Healthcare, DA Best, Netherlands).

Coronary angiographic evaluation: CAG was performed through femoral or radial artery access (Judkins technique). Two cardiologists evaluated the CAG images individually. The SYNTAX score (SS) was calculated by including the vessels with a diameter larger than 1.5 mm and a stenosis over 50% from CAG images (http://www.Syntaxscore.com).

Statistical analysis: The variables were divided as categorical and continuous variables. The Kolmogrov-Smirnov test was used to calculate if continuous variables comply with normal distribution. The variables complying with normal distribution were shown by mean and standard deviation; and non-normally distributed variables were shown by median and interquartile range (IQR). The categorical data were shown in numbers and percentages. A correlation analysis was performed between SS and continuous variables. The variables were shown by Pearson and Spearman correlation coefficients. Linear regression analysis was performed with significant variables in correlation analyses. The independent determinants were detected for SS. The statistical analyses were performed by SPSS 20.0 (SPSS Inc., Chicago, IL) in Windows operating system; *P* < .05 was accepted significant.

## 3 Results

**Figure 1:**
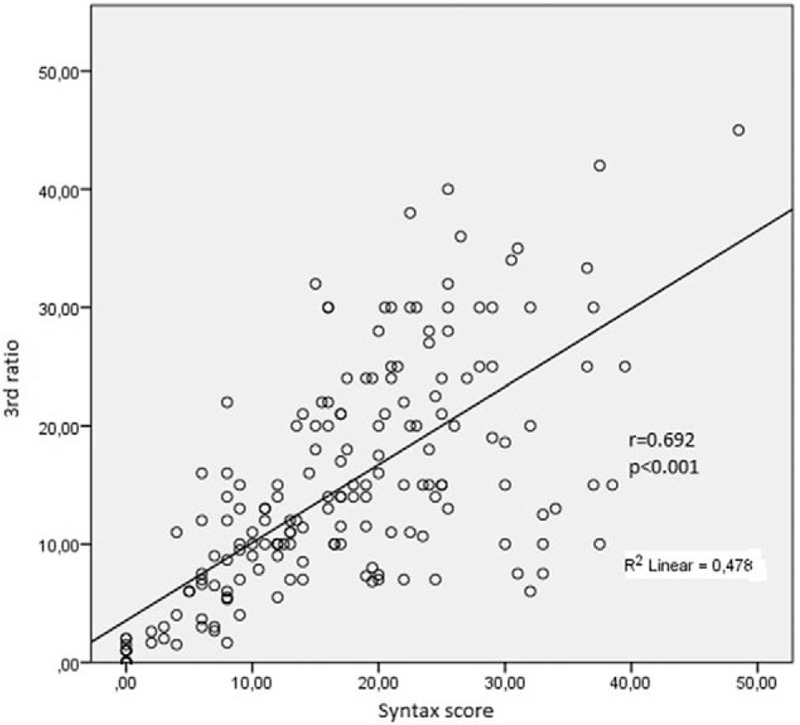
Showing strong correlation between SYNTAX score and third ratio.

We included 306 patients in our study. The age average was 63.1 ± 6.8 and 53.4% of the patients were male. Demographic data of the patients were presented in Table 1. Median hs-TnT was measured 40.9 ng/mL (IQR: 346.4), median CK-MB was measured as 2.9 ng/mL (IQR: 7.5) and the others laboratory findings were shown in Table 2. Mean EF was measured as 53.1 ± 8.3 and it was shown with electrocardiographic data in Table 3. Angiographic data revealed that almost half of the patients (45.1%) had single-artery disease; mean SS was measured as 14.8 ± 9.0 (Table 4). In analysis of bivariate correlation between SS and other variables; a positive-poor correlation with age (*r* = 0.333, *P* < .001), body mass index (*r* = 0.221, *P* = .006), white blood cell (*r* = 0.138, *P* = .02), neutrophile-lymphocyte ratio (*r* = 0.151, *P* = .011), hs-TnT (*r* = 0.230, *P* < .001), CK-MB (*r* = 0.144, *P* = .033); positive-strong correlation with ratio 3 (*r* = 0.692, *P* < .001) and a negative-poor correlation by lymphocyte (*r* = −0.242, *P* < .001), and EF (*r* = 0.312, *P* < .001) (Table 5). Only ratio 3 was determined to be independent predictor for SS in linear regression analysis (OR: 0.642, 95% CI: 0.432–0.853, *P* = .001) (Table 6, Fig. 1).

**Table 1:**
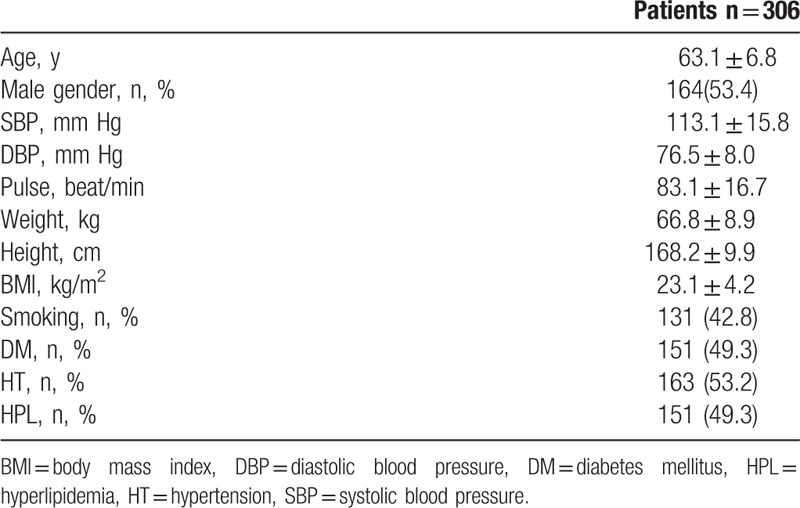
Demographic findings of patients.

**Table 2:**
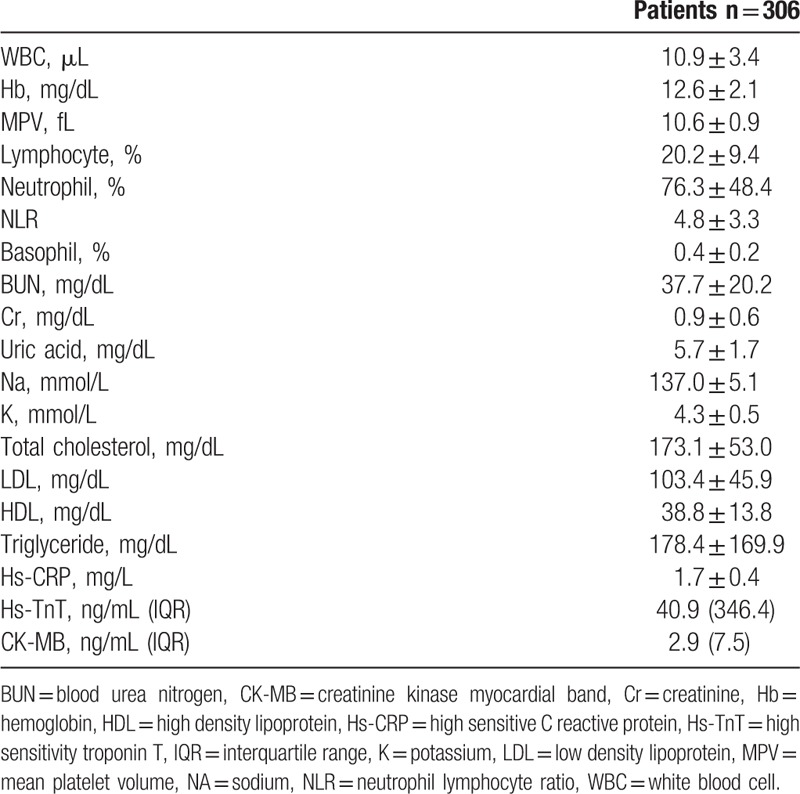
Laboratory findings of patients.

**Table 3:**
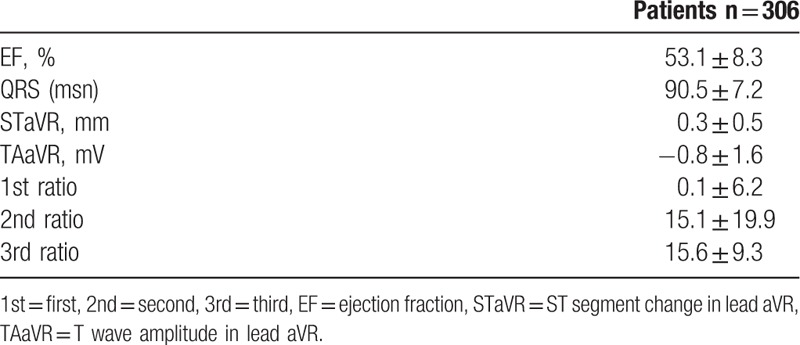
Electrocardiographic and echocardiographic findings of patients.

**Table 4:**
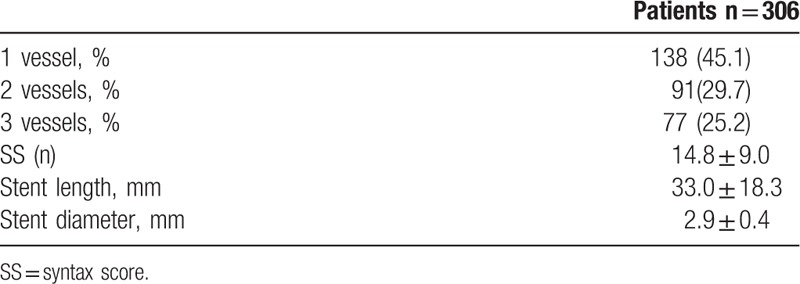
Angiographic findings of patients.

**Table 5:**
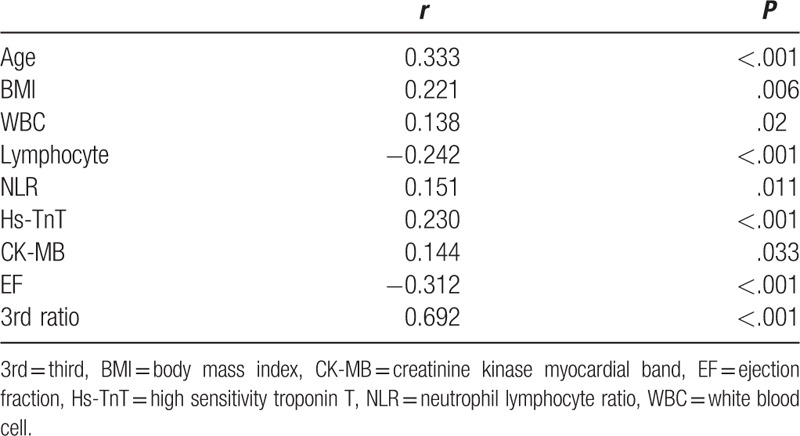
Correlation analysis for SYNTAX score.

**Table 6:**
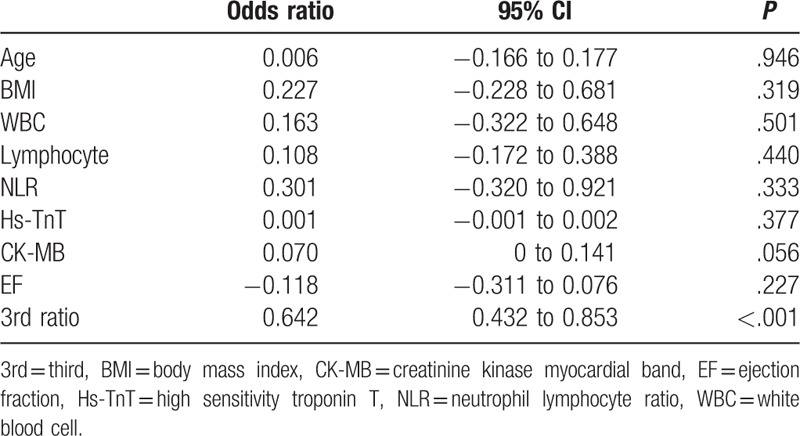
Linear regression analysis for SYNTAX score.

## 4 Discussion

The main finding is independent and strong association between severity of CAD and third ratio in lead aVR in our study. This finding revealed that proportional combination between SS and ECG. This method is simple and inexpensive. It may be calculated by cardiologist or non-cardiologist physician in the ED.

Evaluation of the ischemic changes at aVR derivation is usually ignored when compared with other derivations.^[9,10]^ Such ischemic changes have 2 possible mechanisms. These include transmural or global ischemia in left main coronary artery (LMCA), proximal left anterior descending artery or dominant basal septum which is supplied by first septal branches.^[11–13]^ Three vessel disease or LMCA occlusion have been considered previously when ST elevation is detected in lead aVR.^[14]^ Although some patients with NSTEMI had 3 vessels diseases, they had not presented any significant positive TAaVR or elevated STaVR in our study. Both STaVR and TAaVR in lead aVR were focused in the present study. Accordingly we detected that the third ratio is obtained by dividing the large absolute value by the smaller value. We think that this ratio may be more useful to predict 3 vessel diseases in patients with NSTEMI.

Two studies reported that aVR derivation should not be ignored and is very useful for acute diagnosis and prognosis of acute coronary syndrome, cardiac syndromes with arrhythmia.^[9,10]^ A previous study carried out on patients with NSTEMI detected a significant increase of mortality in the patients with positive TAaVR in low and moderate risk group (troponin negative, pulse < 110 min, systolic blood pressure >90 mm Hg) and no difference in the high risk group. Furthermore, it was suggested that a positive TAaVR is an independent indicator during admission of the patients and for determination of 30-day mortality. They mentioned that a positive TAaVR would help early diagnosis of the patients with high risk for mortality within 30 days. Depending on all conclusions made above, the ST change in lead aVR would be used in addition to TIMI risk scoring system and reclassification of cardiovascular risk.^[15,16]^ Since the present study was designed on a prospective basis, no prognosis was mentioned and no grouping was done. Like previous studies, we did not search positivity of TAaVR only, but also determined a proportional combination from both TAaVR and STaVR ratio by analyzing the ischemic changes in lead aVR as a whole and found the association with SS indicating the severity of CAD. The patients with higher third ratio values might have had an increased mortality risk if they have not been revascularized like the patients with higher SS values. We think that this third ratio can be useful when added to risk score systems (eg, GRACE, TIMI). ECG findings of positive TAaVR and STaVR elevation disappear when the events triggering acute ischemia decrease in some patients. A previous study reported that the patients with ongoing ST segment elevation had a worse prognosis than those who had non-continuous ST elevation.^[17]^ The further status of the ischemic changes was not investigated in the present study; and control ECG findings were not controlled since it was not based on monitoring. If we have reviewed the ECGs taken later on, we could have not found such a strong correlation with SS. Therefore, we believe that the ECGs taken at moment of referral would be more valuable.

SS is a scoring system which provides information about incidence and severity of CAD and allows for a prediction about prognosis and mortality.^[4,18]^ It was observed in a previous study conducted with more patients that annual deaths from all causes, cardiac death and need for revascularization of the target artery increased by increase of SS.^[18]^ There are studies indicating that SS is higher in the high risk group in current scoring system.^[19,20]^ Some studies detected higher SS levels in the elder patients with diabetes, bad renal functions, and NSTEMI.^[21–23]^ The average age of the patients in the present study was <65; renal functions were at the upper limit and half of them were diabetic. Other findings except the age are consistent with previous studies. GRACE and TIMI scoring systems are time-consuming methods which include all risk factors of the patients and are not easy to calculate at a glance. The proportional combination found in the present study is a simple and inexpensive mathematical procedure that may be done by any physician. If such combination is supported by multicentered studies conducted on more patients, it may be included into risk scoring systems and used for admission of the patients before CAG and long-term prognosis estimation.

## 5 Limitations

In the similar studies, the patients had rest before ECG. We have used the ECGs taken at referral to the emergency room. Ischemic is stimulated more in tachycardic events and this triggers the ST change in aVR derivation. This may increase the number of false-positive patients. Multicentered and controlled studies on more number of patients are needed to support the strong correlation and independent determination.

## 6 Conclusion

TAaVR and STaVR ratio in lead aVR on surface ECG may predict severity of CAD in NSTEMI patients. Patients may refer to CAG unit earlier without waiting for cardiac enzymes results.
